# Phorbol esters induce PLVAP expression via VEGF and additional secreted molecules in MEK1‐dependent and p38, JNK and PI3K/Akt‐independent manner

**DOI:** 10.1111/jcmm.13993

**Published:** 2018-11-05

**Authors:** B. JoNell Hamilton, Dan Tse, Radu V. Stan

**Affiliations:** ^1^ Department of Biochemistry and Cell Biology Geisel School of Medicine at Dartmouth Lebanon New Hampshire; ^2^ Norris Cotton Cancer Center Geisel School of Medicine at Dartmouth Lebanon New Hampshire; ^3^ Department of Pathology Geisel School of Medicine at Dartmouth Lebanon New Hampshire

**Keywords:** cancer, caveolae, diapedesis, endothelial diaphragm, fenestrae, inflammation, permeability, transendothelial channel, VVO

## Abstract

Endothelial diaphragms are subcellular structures critical for mammalian survival with poorly understood biogenesis. Plasmalemma vesicle associated protein (PLVAP) is the only known diaphragm component and is necessary for diaphragm formation. Very little is known about PLVAP regulation. Phorbol esters (PMA) are known to induce de novo PLVAP expression and diaphragm formation. We show that this induction relies on the de novo production of soluble factors that will act in an autocrine manner to induce PLVAP transcription and protein expression. We identified vascular endothelial growth factor‐A (VEGF‐A) signalling through VEGFR2 as a necessary but not sufficient downstream event as VEGF‐A inhibition with antibodies and siRNA or pharmacological inhibition of VEGFR2 only partially inhibit PLVAP upregulation. In terms of downstream pathways, inhibition of MEK1/Erk1/2 MAP kinase blocked PLVAP upregulation, whereas inhibition of p38 and JNK MAP kinases or PI3K and Akt had no effect on PMA‐induced PLVAP expression. In conclusion, we show that VEGF‐A along with other secreted proteins act synergistically to up‐regulate PLVAP in MEK1/Erk1/2 dependent manner, bringing us one step further into understanding the genesis of the essential structures that are endothelial diaphragms.

## 1 INTRODUCTION

Endothelial diaphragms^1^, ^2^ are ~40‐80 nm subcellular structures critical for life.^3^, ^4^, ^5^, ^6^, ^7^, ^8^ They occur in endothelial cells (EC) of capillaries and venules in select vascular beds (reviewed in^2^). The diaphragms are thin protein barriers associated with endothelial specific microdomains (ie fenestrae, transendothelial channels, caveolae and vesiculo‐vacuolar organelles) with roles in the maintenance of vascular permeability, blood/tissue homeostasis^3^, ^6^ and the immune function.^5^, ^9^, ^10^, ^11^

Plasmalemma vesicle associated protein (PLVAP or PV1)^12^ is the only known molecular component of the diaphragms^13^, ^14^ and at cellular level, formation of diaphragms is the only function so far demonstrated for PLVAP.^15^ PLVAP knockdown in human^16^ or mouse^17^ cells results in endothelial diaphragms disappearance. Similarly, PLVAP deletion in mice^3^, ^4^, ^5^ or nonsense mutations in humans^6^, ^7^ lead to no diaphragm formation. The in vivo loss of diaphragms results in failure of endothelial barrier function. Leakage of the plasma components into the interstitium of organs with fenestrated capillaries results in protein losing enteropathy, hypoproteinemia and hypertriglyceridemia causing a kwashiorkor‐like wasting syndrome and death.^3^, ^6^

Interestingly, PLVAP reconstitution in the endothelial compartment in mice restores diaphragms exclusively in EC in vascular beds where the diaphragms are native, demonstrating that additional factors are required for diaphragm formation.^3^

Very little is known on PLVAP and diaphragm regulation despite their importance. Phorbol esters such as phorbol myristate acetate (PMA) are known to induce robust de novo formation of fenestrae and transendothelial channels with their associated diaphragms in primary EC in culture.^18^ PMA also induces diaphragms of caveolae and PLVAP expression in MEK1‐dependent and PKC‐independent manner.^16^ Of note, PMA is also a known secretagogue in human EC.^19^

Vascular endothelial growth factor‐A (VEGF‐A) was also shown to be essential for formation and maintenance of fenestrae with diaphragms. VEGF‐A (and not FGF‐2 or VEGF‐C) induces new vessels with fenestrae with diaphragms^20^, ^21^, ^22^, ^23^, ^24^, ^25^ in Rac1 dependent manner.^22^ Deletion of VEGF‐A in the kidney podocytes, pancreas epithelial cells or hepatocytes^26^, ^27^, ^28^ or systemically delivered VEGFR2 inhibitors^29^ result in loss of fenestrae in mice. While overwhelmingly clear in the case of fenestrae, the effect of VEGF‐A/VEGFR2 signalling on PLVAP expression modulation seems to be context dependent. While a VEGFR2 receptor‐selective engineered form of VEGF‐A up‐regulates PLVAP expression in single donor human umbilical vein EC (HUVEC),^30^ primary EC cultured in presence of VEGF‐A do not or poorly express PLVAP^16^ and VEGF‐A has no effect^17^ or even decreases^31^ PLVAP expression in immortalized mouse EC lines that constitutively express PLVAP. Moreover, VEGFR2 signalling inhibition in vivo does not modify PLVAP expression in the lung.^32^

Finally, the downregulation of PLVAP in specialized vascular beds forming the blood‐brain barrier,^33^, ^34^, ^35^, ^36^ or in developing arteries, glomeruli and cell culture^37^, ^38^, ^39^, ^40^ appear to be controlled by the Wnt and Notch signalling pathways respectively.

In order to arrive at a cell culture system where fenestrae with diaphragms and PLVAP could be induced at high frequency by physiological cues in primary EC, we have sought to dissect the molecular mechanism of PLVAP upregulation by PMA. We show that PMA upregulation of PLVAP mRNA and protein depends on de novo protein synthesis and secretion of a group of proteins that act synergistically in autocrine fashion. Among these secreted proteins, we identified VEGF‐A signalling through VEGFR2 as important but not sufficient for PLVAP expression. In addition, we show that PLVAP upregulation by the PMA‐induced secreted factors is MEK1‐dependent and JNK‐, p38‐, PI3K‐ and Akt‐independent.

## 2 MATERIALS AND METHODS

### 2.1 Materials

Recombinant human VEGF‐A was from R&D Systems (Minneapolis, MN) (cat#293‐VE) or PreproTech (Rocky Hill, NJ), (cat#100‐20); Heparin‐Sepharose beads from GE Healthcare (Piscataway, NJ); PMA (cat# P8139) and cycloheximide (CHX) (cat# C4859) from Sigma (St. Louis, MO); pharmacological inhibitors, see Table 1, from EMD Calbiochem (San Diego, CA) or SelleckChem (Houston, TX). All general reagents were from Thermo‐Fisher (Pittsburgh, PA), unless otherwise stated.

**Table 1:** Pharmacological inhibitors used in the study

Name	Concentration used	Target selectivity (IC50 in cell‐free assays)	Other known targets
VEGFR inhibitors			
Axitinib	1‐10 μmol/L	VEGFR1 (0.1 nmol/L), VEGFR2 (0.2 nmol/L), VEGFR3 (0.2 nmol/L)	PDGFRβ and c‐Kit
Cabozantinib (XL184)	0.01‐10 μmol/L	VEGFR2 (0.035 nmol/L)	c‐Met, Ret, Kit, Flt‐1/3/4, Tie2, AXL
Vandetanib (ZD6474)	0.01‐10 μmol/L	VEGFR2 (40 nmol/L), VEGFR3 (110 nmol/L)	EGFR
SAR131675	1‐10 μmol/L	VEGFR3 (23 nmol/L), VEGFR1 (1 μmol/L), VEGFR2 (200 nmol/L)	
MEK1/2 inhibitors			
U0126	0.01‐10 μmol/L	MEK1/2 (70/60 nmol/L)	
PD98059	2.5‐25 μmol/L	MEK1 (2 μmol/L)	
p38 inhibitors			
SB203580	1‐10 μmol/L	p38α/β (300‐500 nmol/L)	Akt
SB202190 (FHPI)	1‐10 μmol/L	p38α (50 nmol/L), p38β (100 nmol/L)	
JNK inhibitors			
SP600125	1‐10 μmol/L	JNK1/2 (40 nmol/L), JNK3 (90 nmol/L)	Aurora A, TrkA, FLT3
Tanzisertib (CC‐930)	1‐10 μmol/L	JNK1 (61 nmol/L), JNK2/3 (5 nmol/L),	ERK1 and p38a
PI3K inhibitors			
Pictilisib (GDC‐0941)	1‐10 μmol/L	PI3Kα (3 nmol/L), PI3Kβ (33 nmol/L), PI3Kδ (3 nmol/L), PI3Kγ (75 nmol/L)	mTOR
Idelalisib (CAL‐101)	1‐10 μmol/L	PI3Kδ (2.5 nmol/L), PI3Kγ (100 nmol/L)	C2β, hVPS34, DNA‐PK and mTOR
Wortmannin	1‐10 μmol/L	pan‐PI3K (3 nmol/L)	DNA‐PK, ATM, MLCK
Akt inhibitors			
Ipatasertib (GDC‐0068)	1‐10 μmol/L	Akt1/2/3 (2/18/8 nmol/L)	

**Table 2:** Gene expression assays (IDT) used for multiplex real‐time PCR

Gene	Assay ID	Fluor	Probe	Forward	Reverse
B2M	Hs.PT.58v.18759587	HEX	5′‐/5HEX/CCTGCCGTG/ZEN/TG AACCATGTGACT/3IABkFQ/‐3′	5′‐ACCTCCATGA TGCTGCTTAC‐3′	5′‐GGACTGGTCTT TCTATCTCTTGT‐3′
PLVAP	Hs.PT.58.39466084	Cy5	5′‐/5Cy5/CCAACCCCC/TAO/AG CCCATCGA/3IAbRQSp/‐3′	5′‐GGATCTTCCTC TTGAACTCCTC‐3′	5′‐TGGACACCT GCATCAAGAC‐3′
VEGF‐A	Hs.PT.58.21234833	FAM	5′‐/56‐FAM/TGCTCTACC/ZEN/T CCACCATGCCAAG/3IABkFQ/‐3′	5′‐GCGCTGATAG ACATCCATGA‐3′	5′‐CCATGAACTTTC TGCTGTCTTG‐3′
VEGFR1	Hs.PT.58.40906831	FAM	5′‐/56‐FAM/CGAGCAGAT/ZEN/T TCTCAGTCGCAGGTA/3IABkFQ/‐3′	5′‐CATCCTCTTCA GTTACGTCCTT‐3′	5′‐GCTCTCTATGAA AGTGAAGGCA‐3′
VEGFR2	Hs.PT.58.3285240	FAM	5′‐/56‐FAM/AGAAGGAGC/ZEN/A ACACACAGTGAGCA/3IABkFQ/‐3′	5′‐GAGGATCTTGA GTTCAGACATGG‐3′	5′‐TTGGAATTGACA AGACAGCAAC‐3′

### 2.2 Antibodies

We used anti‐human PLVAP mAb (clone PAL‐E)^6^ and chicken anti‐human PLVAP‐C pAb^41^; mouse anti‐GAPDH (clone 1E6D9, cat#60004‐1‐Ig, Proteintech, Rosemont, IL), mouse anti‐ACTB mAb (clone AC40; Sigma); rabbit anti‐human VEGF (clone 500‐P10, PeproTech and clone A‐20 Santa Cruz Biotechnology, Dallas, TX); mouse anti‐human VEGF (Ab‐1) from Neomarkers/Thermo Scientific (Pittsburgh, PA); phospho‐specific antibodies against MAP kinases (Erk1/2, p38, JNK) and Akt from Cell Signaling Technologies (Danvers, MA); IRDye680 and IRDye800‐secondary antibodies were from LiCOR (Lincoln, NE); HRP‐conjugated goat anti‐rabbit IgG‐HRP, rabbit anti‐chicken IgG‐HRP and goat anti‐mouse IgG‐HRP from Biodesign (Saco, ME).

### 2.3 Cells and cell culture

Human umbilical vein EC and neonate dermal microvascular EC (HDMVECn) were obtained from Lonza or PromoCell (Heidelberg, Germany) and were cultured according to manufacturer's instructions. HUVEC were cultured in EGM2, HDMVECn were cultured in EGM2‐MV (Lonza) or ECGM‐MV2 (PromoCell) medium. Cell culture certified bovine serum albumin (BSA) solution was purchased from Millipore‐Sigma (St. Louis, MO) (cat# A9576).

### 2.4 PMA treatments

Endothelial cells were seeded at 50%‐80% confluence in 1% gelatin (Sigma, cat# G9391)—coated culture plates and grown to near confluence for 24‐48 hours in the manufacturer recommended growth media. Unless otherwise noted, EC were rinsed, and serum starved (2 hours, 37°C, 5%CO2) in EC basal medium (EBM2 or ECBM‐2) prior to PMA treatment.

#### 2.4.1 Chronic PMA treatment

Endothelial cells were treated (37°C, 5% CO_2_) with 50 nmol/L PMA in EBM2 supplemented with 5% heat inactivated FBS (EBM‐FBS). After noted amounts of time, the PMA containing medium was aspirated, the cells rinsed twice in DPBS and processed for protein or RNA analysis.

#### 2.4.2 Pulsed PMA treatment

Cells were “pulse stimulated” for 30 minutes (37°C, 5%CO_2_) with noted concentrations of PMA in 2% BSA in EBM2 (EBM‐BSA), the medium aspirated, the cells rinsed (2x, RT) with EBM2 and chased for the indicated time points. The chase medium consisted of either EBM‐BSA, EBM‐FBS or full growth medium. At indicated time points, supernatant and cells were harvested and further processed for protein or RNA analysis.

### 2.5 Protein synthesis inhibition with cycloheximide

For chronic PMA and for conditioned medium (CM) treatments, EC were seeded in duplicate on gelatin‐coated plates, grown to near confluence, serum starved 1.5 hours in EBM2 and 30 minutes presence of 10 μg/mL CHX in EBM‐BSA and stimulated for the duration of the experiment with 50 nmol/L PMA + 10 μg/mL CHX or with 4‐6 hours CM + 10 μg/mL CHX. For pulsed PMA treatment, the difference was that EC were stimulated with PMA/CHX for only 30 minutes followed by chase in EBM‐FBS containing 10 μg/mL CHX. At indicated time points, cells were rinsed twice in DPBS and lysed for RNA or protein analysis.

### 2.6 Conditioned medium treatments

For clarity, schematics of the experimental design are presented in the respective figures. Human EC (HUVEC or HDMVECn), labelled donor EC, were seeded at 70% density into gelatin‐coated six well plates and grown for 24 hours before serum starvation (2 hours, 37°C, 5% CO_2_, 2 mL per well) in EBM2 basal medium and pulse treatment (15 minutes, 37°C) with 1.5 mL per well of 50 nmol/L PMA or vehicle (DMSO) in EBM‐BSA. The drug was washed away with EBM2 medium (2x, RT, 2 mL per well), followed by donor EC incubation (37°C, 5% CO_2_, 1.5 mL per well) with EBM‐FBS. At the indicated time points, the EC CM containing EC secreted factors was collected and the donor cells further incubated (37°C, 5% CO_2_) in EBM‐FBS. At 24 hours after PMA treatment the donor cells were washed in DPBS and lysed for protein or RNA analysis.

The CM from donor EC was immediately transferred onto PMA naive, serum starved (2 mL EBM2 per well, 2 hours, 37°C, 5% CO_2_), confluent acceptor EC grown on gelatin‐coated six well plates. Acceptor cells were incubated (37°C, 5% CO_2_) with CM for 24 hours, washed twice in DPBS and lysed for protein or RNA analysis.

#### 2.6.1 Heparin depletion of conditioned medium

Conditioned medium peaks (4‐6 and 6‐8 hours) were collected from donor cells cultured in six well plates. For each peak the respective CM was pooled in a 15 mL tube and split into two halves. One half was left untreated (control), the other half of the volume was added to 1 mL settled gel of heparin‐agarose previously equilibrated (3×, 5 minutes, RT) in EBM‐BSA. The mixture was further incubated (1 hour, RT) with gentle end‐over‐end rotation before the beads were pelleted by centrifugation (600 *g*, 10 minutes, RT). Two mL per well control CM or heparin‐depleted CM was added to serum starved (2 hours, 37°C, 5% CO_2_) acceptor cells grown in duplicate wells in a six well plate, incubated (37°C, 5% CO_2_) for 24 hours when the acceptor cells were washed twice with DPBS and directly lysed in 200 μL per well of non‐reducing sample buffer. Equal volumes of the lysate were resolved by 8% SDS‐PAGE and subjected to immunoblotting with anti‐PLVAP and ACTB antibodies.

#### 2.6.2 Heat inactivation of CM

Conditioned medium “peaks” were collected after PMA treatment and split into two equal volumes: one half was heat inactivated (45 minutes, 60°C followed by 2 minutes on ice) and the other one left untreated (control) before transfer to serum starved acceptor cells.

#### 2.6.3 Pertussis toxin treatment

Acceptor cells were serum starved and then treated for 24 hours with CM in presence or absence of 0.1 μg/mL pertussis toxin (PT) (Sigma, cat# P7208).

#### 2.6.4 CM fractionation by ultracentrifugation

Conditioned medium “peaks” (4‐6 hours) were collected and subjected to ultracentrifugation (1 hour, 100 000 *g*, 4°C). The supernatant (S), containing the soluble factors, was transferred to serum starved acceptor cells and further incubated for 24 hours (37°C, 5% CO_2_). The pellet (P), containing any particulate matter (exosomes) was resuspended in an equivalent volume of EBM‐FBS by pipetting and vortexing and transferred to donor cells.

### 2.7 Inhibition of VEGF‐A signalling using anti‐VEGF‐A antibodies

Conditioned medium “peaks” (4‐6 and 6‐8 hours) containing EC secreted factors were collected after PMA treatment. The CM peaks adjusted to 0.1 or 1 μg/mL of control or anti‐VEGF antibodies, incubated (1 hour, RT) with gentle rotation, added to PMA naive acceptor EC which were further cultured (24 hours, 37°C, 5% CO_2_) and collected for protein isolation and WB with anti‐PLVAP and ACTB antibodies.

### 2.8 Pharmacological inhibition of downstream signalling of PMA and PMA conditioned medium

Endothelial cells were seeded in six well plates, grown (24 hours, 37°C, 5% CO_2_) to confluence in full growth medium, serum starved for 90 minutes in EBM2, treated with the inhibitors in EBM2 for 30 minutes before adding PMA ± inhibitors in EBM‐BSA and chased in EBM‐FBS for indicated times when cells were rinsed and processed for RNA or protein analysis. The specificity, IC50 and concentration range tested for each inhibitor are given in Table 1. All the inhibitor stocks were freshly made as 1000× working stocks in EBM2 right before the experiment.

Conditioned medium peaks were collected, adjusted to the final concentrations of different inhibitors and added to serum starved naive acceptor EC that were preincubated (10 minutes, 37°C, 5% CO_2_) with the same concentration of the respective inhibitors. After the indicated times, acceptor cells were collected for RNA or protein isolation. For immunoblotting, cells were solubilized (5 minutes, 100°C) in SDS‐PAGE sample buffer containing 2% beta‐mercaptoethanol and 1 mmol/L NaVO_4_. The phosphorylation status of relevant enzymes was determined with the respective phospho‐specific antibodies.

### 2.9 CM/CM experiments

4‐6 hours CM was generated from donor cells and used in a pulse experiment (30 minutes, 37°C, 5% CO_2_) on serum starved (2 hours, 37°C in EBM2) naive acceptor cells. After incubation, the 4‐6 hours CM was aspirated from acceptor cells, the cells rinsed 2× with room temperature EBM2 and the cells chased in EBM‐FBS. Every 2 hours (ie at 2, 4, 6 and 8 hours) the 4‐6 hours CM conditioned medium (labelled conditioned medium/conditioned medium [CM/CM] medium) was collected and replaced with fresh EBM‐FBS. The peaks thus collected (ie 0‐2, 2‐4, 4‐6 and 6‐8 hours CM/CM) were immediately transferred to serum starved (2 hours, 37°C in EBM2) naive HDMVECn and incubated for 24 hours when the cells were collected for PLVAP mRNA level evaluation by real‐time PCR.

### 2.10 Multiplex cytokine assays

Cytokines were measured in CM using Bio‐Plex human cytokine multiplex kits (Bio‐Rad, Hercules, CA) by DartLab core facility at Dartmouth.

### 2.11 RNA isolation

Total RNA was isolated using RNAeasy mini kit (Qiagen, Germantown, MD) or Quick RNA Mini‐prep Kit (Zymo Research, Irvine, CA), as per manufacturer's instructions. RNA integrity and quality were determined using Bioanalyzer (Agilent, Santa Clara, CA) and NanoDrop (Thermo‐Fisher). RNA preps with *A*260/280 ratios ranging from 1.97 to 2.02 were used.

### 2.12 Real‐time quantitative PCR

Quantitative real‐time PCR was done as before.^15^ Briefly, 2 μg of total RNA was reverse transcribed using High Capacity cDNA Reverse Transcription Kit (Applied Biosystems, Foster City, CA). Real‐time PCR employing TaqMan Gene Expression Master Mix and Gene Expression Assays was carried out on an ABI 7500 RT‐PCR system (Applied Biosystems). The gene expression assays used were: PLVAP (Hs00229941_m1), VEGF‐A (Hs00900055_m1), VEGFR1 (Hs01052961_m1), VEFR2 (Hs00911700_m1) and ACTB (Hs03023880_g1). The reactions were performed in triplicate, utilizing cDNA corresponding to 10 ng RNA input. Cycling conditions were as follows: cycle 1: step 1—50°C for 2 minutes, step 2—95°C for 10 minutes; cycles 2–40: step 1—95°C for 15 seconds and step 2—60°C for 1 minute.

For multiplex quantitative real time PCR, reverse transcription was performed on 200 ng RNA using QuantiNova Kit (Qiagen). Triplex qPCR was performed in duplicate using Luna Universal Probe qPCR Master Mix (New England Biolabs, Ipswich, MA) to determine levels of PLVAP, VEGF, VEGFR1 and VEGFR2 mRNA (Table 2). Human beta‐2‐microglobulin (B2M) was used as a housekeeping gene. PCR was run on BioRad CFX96 thermal cycler performed with the following conditions: cycle #1—step 1 95°C for 10 minutes; cycles #2‐40—step 1: 95°C for 15 seconds, step 2: 60°C for 1 minute.

Gene expression was quantitated performed with both absolute and relative methods, as described.^42^ For absolute quantification, the PCR signal of each gene was compared to the signal obtained using standard curves generated using five 10‐fold serial dilutions (100 000 to 10 copies) of constructs containing the gene expression assay target sequence cloned into the pGEM‐T vector (Promega, Madison, WI). For each gene, the copy number × Ct (threshold cycle) was plotted and the curve used to calculate the copy number in each sample. In addition, fold difference over the non‐treated control (NTC) was calculated as follows: FD (fold difference) = 2^−ΔΔCt^ in which ΔΔCt = ΔCt−(4 hours NTC ΔCt) and ΔCt = (target RNA Ct)−(calibrator Ct) where calibrator was either ACTB or B2M.

### 2.13 RT‐PCR for VEGFA isoforms

Total RNA (200 ng) from control and PMA treated HDMVECn was reverse transcribed and human VEGF‐A isoforms were amplified using forward (5′‐TGCGGATCAAACCTCACCAA‐3′) and reverse (5′‐CCTCCGGACCCAAAGTGCT‐3′) primers located in exons 4 and 8b of the human VEGF‐A gene, respectively. Primer sequences and PCR amplification conditions were as described.^43^ The expected PCR amplicon sizes were: 319 nucleotides for VEGF‐A‐121 (NCBI transcript NM_001025370.2), 451 nucleotides for VEGF‐A‐165 (NCBI transcript NM_001025368.2), 523 nucleotides for VEGF‐A‐189 (NCBI transcript NM_001171624.1).

### 2.14 Western blotting

Cells were placed on ice, rinsed twice with ice‐cold PBS, and lysed in 10 mmol/L Tris‐Cl, pH 6.8 containing 0.5% SDS and protease inhibitors cocktail (P8340—Sigma‐Aldrich). Detergent extracts were cleared by centrifugation (10 minutes, 16°C, 16 000 *g*) and protein concentration determined using the bicinchoninic acid protein assay (Thermo‐Fisher). Equal amounts (20 μg per lane) of total EC proteins were heated 5 minutes at 100°C in SDS‐PAGE sample buffer, resolved by SDS‐PAGE and electro‐transferred to PVDF membrane (Millipore‐Sigma). In some experiments, cells were lysed directly in SDS‐PAGE non‐reducing sample buffer and processed as above.

PVDF membranes containing EC proteins were blocked (30 minutes, RT) with blocking buffer (LiCOR), incubated (overnight, 4°C) with primary antibodies diluted in blocking buffer, washed (3 × 5 minutes, RT) in PBS, and incubated (30 minutes, RT) with either HRP‐ or IRDye‐680 or ‐800 (LiCOR) labelled secondary antibodies. For HRP‐labelled secondary antibodies, the signal was generated using SuperSignal West chemiluminescent substrates (Thermo‐Fisher) and images acquired using a G:Box Chemi XT16 imaging system and GeneSnap software (Syngene, Frederick, MD). The near infrared signal of the IRDye‐labelled antibodies was detected with an Odyssey fluorescence scanner (LiCOR).

Equal loading of lanes was confirmed by blotting membranes with antibodies against housekeeping genes such as of mouse anti‐ACTB mAb (clone AC40) and mouse anti‐GAPDH mAb (clone 1E6D9). Signal quantitation by densitometry on TIFF files was carried out using GelEval v1.35 software (FrogDance, UK) or ImageStudio Lite (LiCOR).

### 2.15 Statistics

Data were analysed using Student's *t* test. *P* < 0.05 was taken as the level of significance.

## 3 RESULTS

### 3.1 Upregulation of PLVAP mRNA by PMA requires protein translation

**Figure 1:**
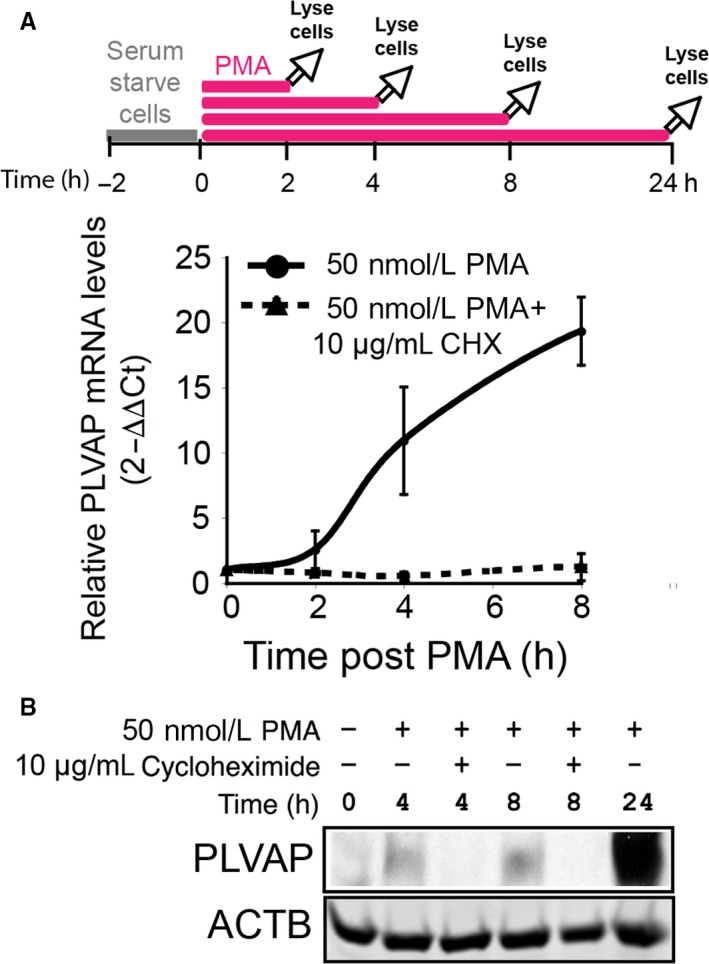
Plasmalemma vesicle associated protein (PLVAP) mRNA upregulation by phorbol myristate acetate (PMA) requires protein synthesis. (A) Relative PLVAP mRNA levels as determined by real time PCR and quantitated using the 2^−ΔΔCt^ method. Total RNA from non‐treated control EC (time 0) or EC treated for 2, 4 or 8 h with 50 nmol/L PMA (solid line) or 50 nmol/L PMA+10 μmol/L CHX (dashed line) were reverse transcribed and probed with validated PLVAP and ACTB Taqman gene assays. (B) Immunoblotting with chicken anti‐human PV1 C pAb (top panel) and anti‐ACTB mAb (lower panel) of EC lysates treated with 50 nmol/L PMA ± 10 μg/mL cycloheximide for 4 or 8 h. EC lysates treated with 50 nmol/L PMA for 24 h were used as positive control for PMA induction of PLVAP

In a first step, we asked whether PMA‐induced PLVAP mRNA transcription depended on de novo protein synthesis. To answer this, we treated primary human HDMVECn with 50 nmol/L PMA (concentration demonstrated to up‐regulate PLVAP and induce the formation of endothelial diaphragms and fenestrae^16^) in presence or absence of CHX, a protein synthesis inhibitor.^44^ As shown previously,^16^ cells were exposed to PMA for the entire duration of the experiment. PLVAP ****mRNA significantly increased in time‐dependent manner starting at ~2 hours after PMA treatment onset (Figure 1A). However, there was no increase of PLVAP mRNA or protein (Figure 1B) when cells were treated with PMA in presence CHX for up to 8 hours of treatment, demonstrating that PLVAP upregulation by PMA requires de novo protein synthesis.

### 3.2 PLVAP is up‐regulated by PMA‐induced soluble proteins

**Figure 2:**
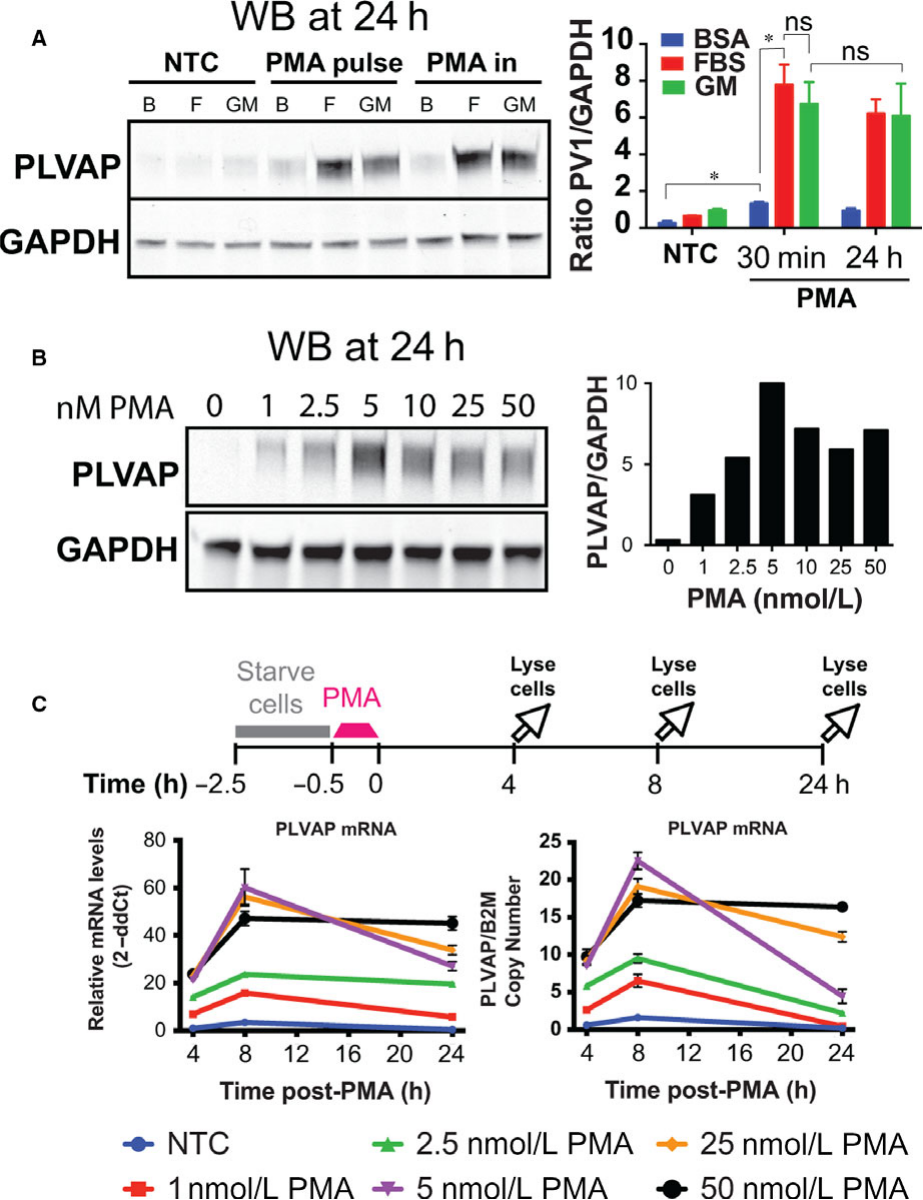
A short pulse of phorbol myristate acetate (PMA) induces plasmalemma vesicle associated protein (PLVAP) mRNA and protein in time‐ and dose‐dependent manner. (A) PMA up‐regulates PLVAP protein in serum dependent manner. Left—Western blotting with anti‐PLVAP and ‐GAPDH antibodies of HDMVEC lysates treated with 50 nmol/L PMA for 30 min or 24 h. The samples were chased or treated, respectively, in EBM‐BSA (B), EBM‐FBS (F) or full growth medium (GM). Right ‐ quantitation of the Western blotting signal (SEM, n > 3, *P < 0.05). For all treatments there was a statistically significant increase in PLVAP levels in PMA treated samples versus NTC and between EBM‐BSA versus EBM‐FBS or GM. No statistically significant difference was found between the two treatments for EBM‐FBS and GM. (B) Immunoblotting with anti‐PLVAP and ‐GAPDH antibodies of HDMVEC lysates treated with the noted concentrations of PMA for 30 min (left) and quantitation of the Western blotting signal (right). (C) Relative PLVAP/B2M mRNA levels induced by different concentrations of PMA. Data are expressed as relative mRNA levels by ΔΔCt method (left) and ratio of mRNA copy numbers (right) relative to beta 2 microglobulin gene (B2M). All PMA treated samples had a statistically significant increase in PLVAP mRNA compared to NTC (SEM, n = 3, *P < 0.05)

We next asked whether the newly synthesized proteins needed to be secreted and possibly acted in autocrine fashion. First, we showed that a 30‐minute pulse of 50 nmol/L PMA followed by its removal and chase using a defined medium elicits similar levels of PLVAP protein at 24 hours post stimulation when compared to 24 hours “chronic” PMA treatment (Figure 2A) with the highest levels of PLVAP protein sustained by EBM‐FBS or EGM as chase medium (Figure 2A). Peak response was observed at 8 hours post pulse at doses ≥5 nmol/L PMA but remained high at 24 hours only for doses of ≥25 nmol/L (Figure 2C). Based on these results, a 30 minutes pulse of 50 nmol/L PMA stimulation of EC and using EBM‐FBS as chase medium was selected for the CM preparation.

**Figure 3:**
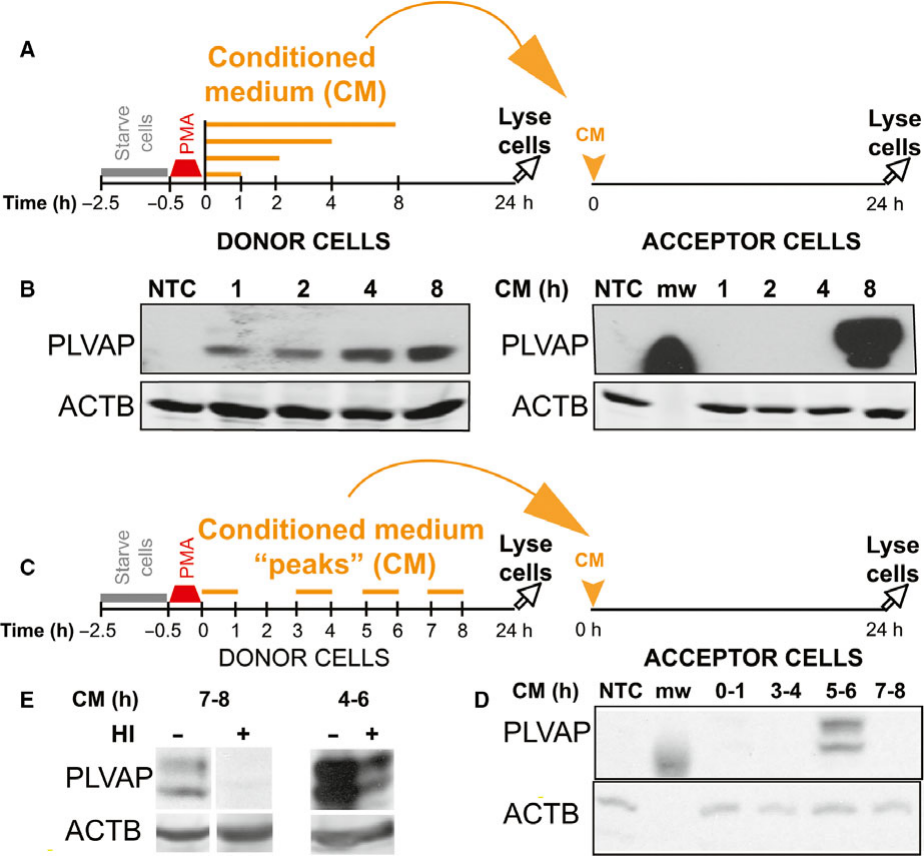
Phorbol myristate acetate (PMA) induces timed secretion of heat sensitive factor(s) that up‐regulate plasmalemma vesicle associated protein (PLVAP) in naive acceptor endothelial cells (EC). (A, B) Schematic of the experimental set‐up (A) and immunoblotting of EC lysates (B) with anti‐PLVAP (top panel) and ACTB (lower panel) antibodies. Donor cells were treated with a pulse of 50 nmol/L PMA and the conditioned medium (CM) was collected after 1, 2, 4, or 8 h and immediately transferred onto naive acceptor EC. (C, D) Schematic of the experimental set‐up (C) and immunoblotting of EC lysates (D) with anti‐PLVAP (top panel) and ACTB (lower panel) antibodies. Donor cells were treated with a pulse of 50 nmol/L PMA and the CM was collected between 0‐1, 3‐4, 5‐6, or 7‐8 h and immediately transferred onto naive acceptor EC which were incubated for 24 h, lysed and subjected to immunoblotting. (E) Heat inactivation of the 4‐6 and 7‐8 hours CM peaks results in sharply decreased PLVAP upregulation. Western blotting with anti‐PLVAP and anti‐GAPDH

Serum starved (2 hours, 37°C, 5% CO_2_) naive acceptor EC (schematic in Figure 3A) were treated (24 hours, 37°C, 5% CO_2_) with CM collected from donor cells at 1, 2, 4, and 8 hours post‐PMA pulse. The 8 hours CM was the most potent in upregulating PLVAP protein in naive acceptor EC (Figure 3B, right) demonstrating that between 4‐8 hours after PMA pulse, donor ECs secrete factors capable of robustly upregulating PLVAP. Heat induced denaturation severely reduced the CM ability to induce PLVAP (Figure 3E), confirming the protein nature of the secreted factors. All donor EC showed the expected PMA‐induced upregulation of PLVAP at 24 hours (Figure 3B, left), further supporting these conclusions.

A more refined analysis in which donor EC were pulsed with PMA, chased in EBM‐FBS and 1 hours CM “peaks” were collected and incubated with naive acceptor EC for 24 hours (Figure 3C), showed that the CM collected between 5 and 6 hours post‐PMA was able to induce a robust upregulation of PLVAP in naive EC, while other time points had minimal effect (Figure 3D). Across multiple experiments carried out with different EC, the highest PLVAP‐inducing “activity” was always found in the CM peaks collected between 4 and 8 hours. However, 4‐6 hours CM was usually more potent than 6‐8 hours CM in upregulating PLVAP.

### 3.3 PMA up‐regulates PLVAP via heparin‐binding and pertussis toxin‐insensitive factors

**Figure 4:**
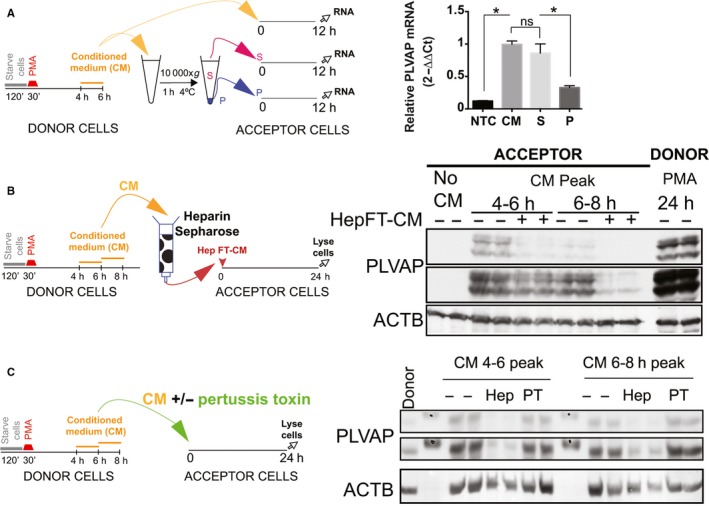
Phorbol myristate acetate (PMA) up‐regulates plasmalemma vesicle associated protein (PLVAP) in part via soluble, secreted, heparin‐binding factors. (A) Schematic of the experimental set‐up (left) and relative PLVAP mRNA levels induced by the CM fractions in acceptor EC at 12 h after treatment (right). S—CM soluble fraction, P—CM particulate pellet. (SEM, n = 3, *P < 0.05). (B) Schematic of the experimental set‐up (left) and immunoblotting with anti‐PLVAP (the two upper panels, representing different exposure times) and anti‐ACTB (lower panel) of acceptor EC lysates after 24 h treatment with 4‐6 and 6‐8 hours CM peaks or the respective CM depleted on heparin‐Sepharose (Heparin +). The last two lanes on the right (PMA 24 h) are duplicates of PMA treated donor cells used to generate the 4‐6 and 6‐8 hours CM peaks. (C) Schematic of the experimental set‐up (left) and immunoblotting (right) with anti‐PLVAP (two upper panels, representing different exposure times) and anti‐ACTB (lower panel) antibodies of acceptor EC lysates after 24 h treatment with 4‐6 and 6‐8 hours CM peaks in absence (−) or presence (PT) of pertussis toxin. For comparison, lysates of acceptor EC treated with CM peaks depleted with heparin‐Sepharose (Hep) were included

To determine whether the secreted factor(s) upregulating PLVAP are soluble or membrane bound (exosomes), we fractionated the 4‐6 h CM into particulate (P) and soluble (S) fractions using ultracentrifugation (see Methods) and tested their ability to up‐regulate PLVAP in acceptor cells (schematized in Figure 4A, left). Both soluble (S) and particulate (P) fractions had the ability to up‐regulate PLVAP mRNA at 12 hours, the soluble fraction was more potent (Figure 4A, right).

To gain insight into the chemical nature of the PLVAP‐inducing soluble factor(s), we depleted CM peaks (4‐6 and 6‐8 hours CM) of heparin‐binding proteins. As shown in (Figure 4B, left), the depletion led to marked decreased in CM ability to induce PLVAP protein in naive acceptor cells (Figure 4B, right). Interestingly, the residual activity could not be eliminated even after passages over two sequential heparin columns, (data not shown).

Endothelial cells produce chemokines, secreted factors that can bind heparin.^45^ We therefore tested the ability of 4‐6 and 6‐8 hours CM peaks to up‐regulate PLVAP in presence of PT, a general/broad spectrum chemokine receptor inhibitor^46^, ^47^ (schematized in Figure 4C, left). Treatment of acceptor EC with PT had no effect on PLVAP protein upregulation by the 4‐6 and 6‐8 hours CM peaks (Figure 4C, right), ruling out a role for chemokine signalling in this system.

### 3.4 PMA up‐regulates PLVAP in part via VEGF/VEGFR2 signalling

Phorbol myristate acetate up‐regulates VEGF‐A a known heparin‐binding growth factor^48^ and its receptors, VEGFR1 and VEGFR2 in HUVEC and HDMVEC,^49^ making it them as good candidates for PLVAP upregulation by PMA in EC.

**Figure 5:**
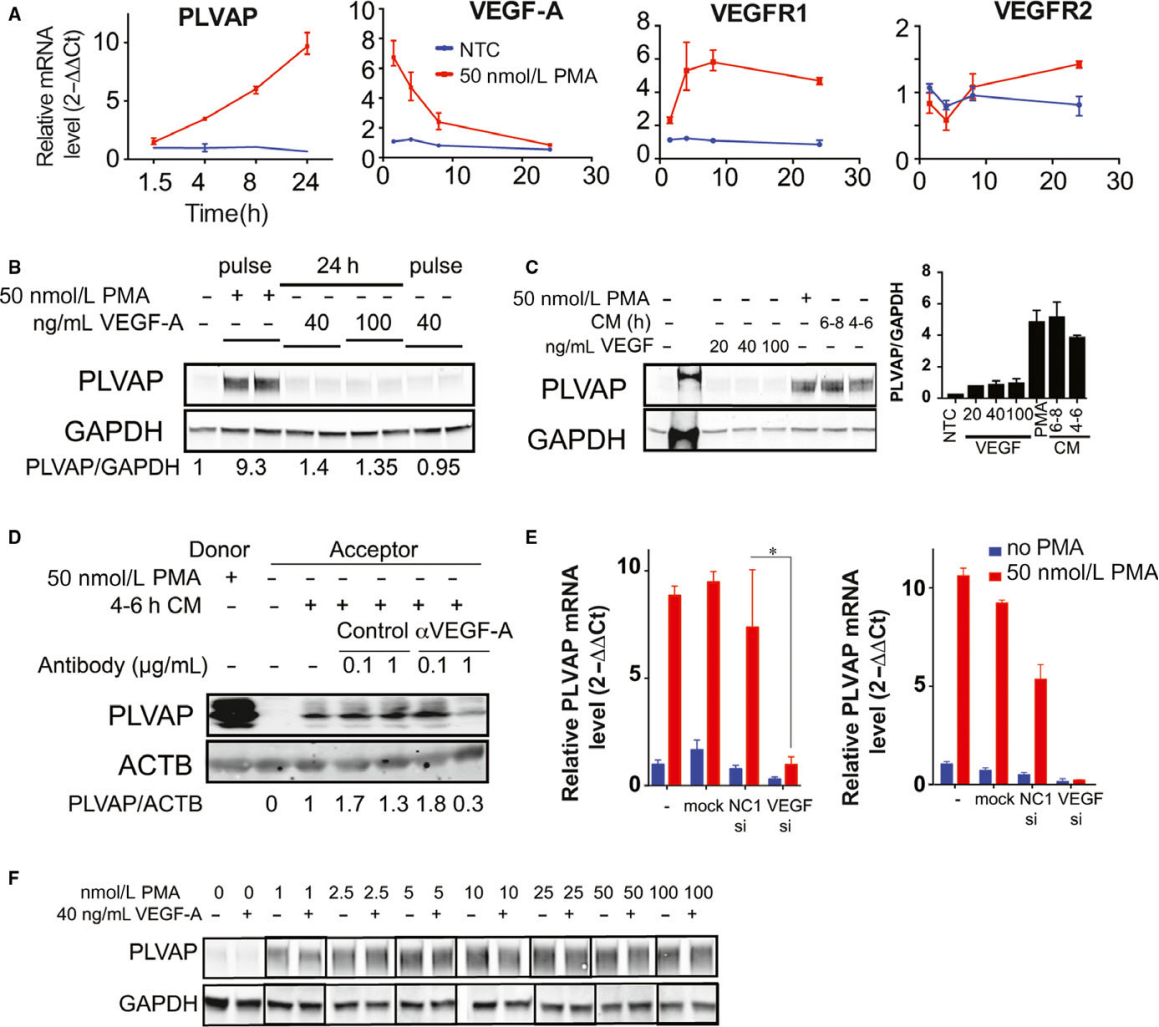
Phorbol myristate acetate (PMA) up‐regulates plasmalemma vesicle associated protein (PLVAP) via vascular endothelial growth factor (VEGF)/VEGFR2 signalling. (A) Time course of PLVAP, VEGF‐A, VEGFR1 and VEGFR2 mRNA changes elicited by a 30 min 50 nmol/L PMA pulse detected by real time PCR (SEM, n > 6, *P > 0.05). (B, C) PMA or CM is more effective than VEGF‐A alone in upregulating PLVAP protein. Western blotting with anti‐PLVAP and anti‐GAPDH antibodies of HDMVECn total cell lysates at 24 h after treatment with 50 nmol/L PMA pulse, the noted doses of VEGF‐A or CM. Right panel in (C)—densitometric quantitation of the signal ratio of PLVAP/GAPDH. (D) Anti‐VEGF‐A antibodies inhibit PLVAP upregulation by 4‐6 hours CM. Western blotting with anti‐PLVAP and anti‐ACTB of acceptor EC total cell lysates. (E) VEGF‐A siRNA inhibits PLVAP mRNA upregulation by PMA. Real time PCR quantitation of PLVAP (left) and VEGF‐A (right) relative mRNA levels (ΔΔCt method) at 8 h post‐PMA or vehicle (“no PMA”) treatment. “‐” non‐transfected control, “mock”—mock transfected cells, no siRNA, “NC1”—negative control siRNA duplex (SEM, n > 3, *P >* *0.05). (F) Combination of VEGF‐A and PMA does not increase PLVAP levels compared to PMA alone. Western blotting with anti‐PLVAP and anti‐GAPDH antibodies of HDMVECn total cell lysates treated with 40 ng/mL VEGF‐A and the noted concentrations of PMA

As seen in Figure 5A, a 30‐minute 50 nmol/L PMA pulse treatment efficiently up‐regulated VEGF mRNAs (VEGF‐A‐121, VEGF‐A‐165 and ‐189 isoforms, Figure S1A) in HDMVEC with a peak at 60‐90 minutes post‐PMA, accompanied by an increase in VEGF‐A protein secretion in the medium at 3 hours as detected by a Luminex assay that does not discriminate between the different VEGF‐A isoforms (data not shown). Increases in VEGFR1 but not VEGFR2 mRNA were also observed in HDMVECn up to 8 hours (Figure 5A). However, the VEGFR2 mRNA levels were already substantial in HDMVEC (1510 ± 110 SEM mRNA copy numbers/ng total RNA, n = 22 samples), as determined by absolute quantitative PCR methods.

A direct comparison of PLVAP protein levels induced by VEGF‐A‐165 (30 minutes or 24 hours treatment) with those induced by PMA or CM (4‐6 and 6‐8 hours) demonstrate PMA (Figure 5B) or CM (Figure 5C) to be much more efficient. VEGF functionality was validated by its effectiveness in inducing VEGFR2 phosphorylation at 2‐5 minutes post VEGF exposure (data not shown).

Next, we treated the 4‐6 hours CM with anti‐VEGF‐A antibodies. Doses of 1 μg/mL of anti‐VEGF‐A antibody resulted in a detectable inhibition of PLVAP protein upregulation by the 4‐6 hours CM (Figure 5D) in acceptor cells as compared to lower (0.1 μg/mL) anti‐VEGF‐A concentrations, irrelevant IgG or non‐treated CM (Figure 5D). Additionally, VEGF‐A mRNA knockdown by siRNA inhibited PLVAP mRNA upregulation by PMA (Figure 5E). However, treatment of EC with up to 40 ng/ml VEGF in addition to PMA does not further increase PLVAP protein levels (Figure 5F), suggesting that VEGF‐A acts downstream of PMA.

**Figure 6:**
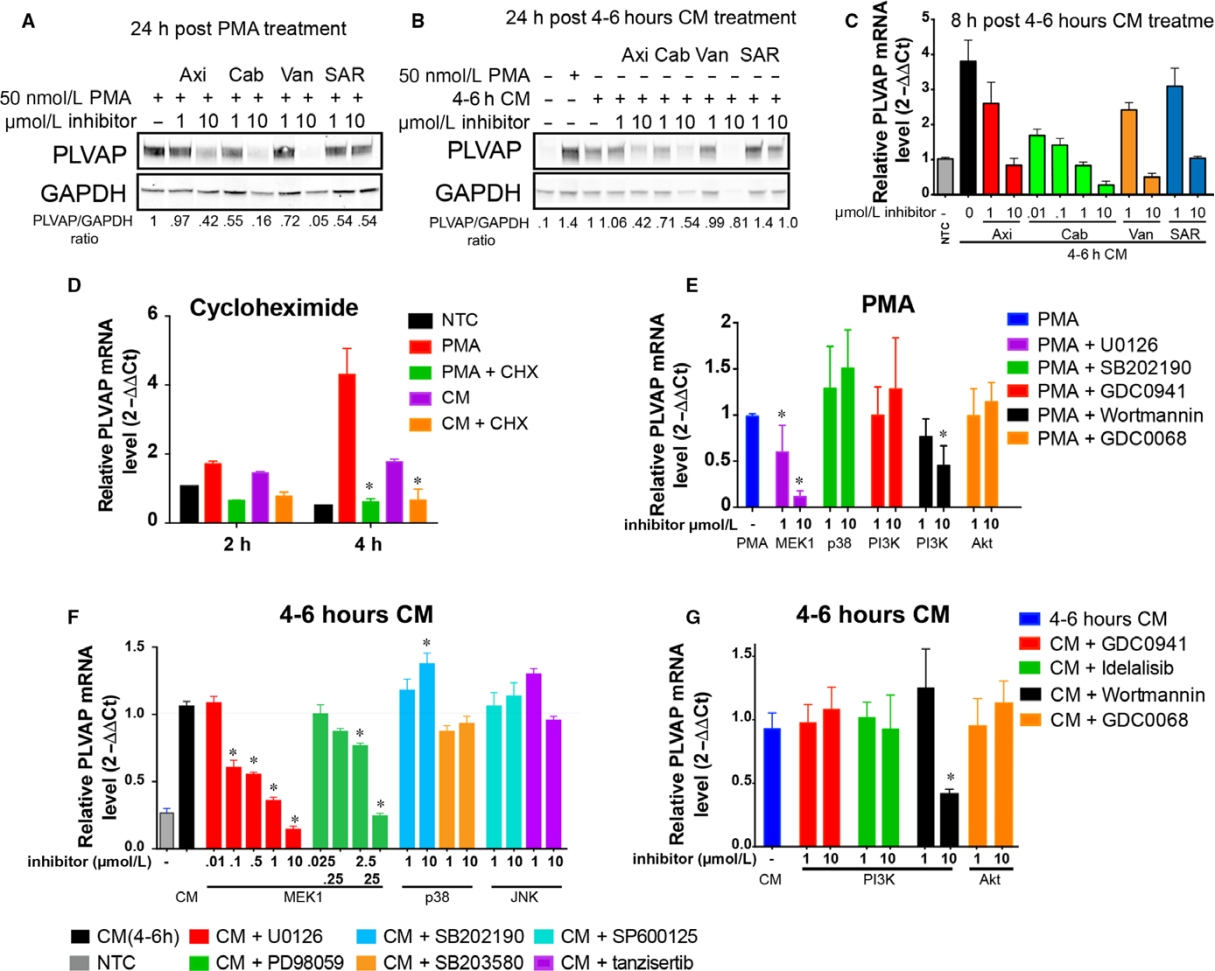
Phorbol myristate acetate (PMA) up‐regulates plasmalemma vesicle associated protein (PLVAP) in VEGFR2‐ and MEK1‐dependent and p38‐, PI3K‐ and Akt‐independent manner. (A, B) Pharmacological inhibitors of VEGFR2 signalling inhibit PLVAP protein upregulation by PMA (A) or 4‐6 hours CM (B). Western blotting with anti‐PLVAP and anti‐GAPDH of HDMVECn total cell lysates. Densitometric quantitation of the signal ratio of PLVAP/GAPDH is noted under the blot. (C) Pharmacological inhibitors of VEGFR2 signalling inhibit PLVAP mRNA upregulation by 4‐6 hours CM. Real time PCR quantitation of PLVAP relative mRNA levels 8 h post CM treatment. (SEM, n = 3, *P > 0.05). (D) PLVAP upregulation by 4‐6 hours CM requires de novo protein synthesis. Relative PLVAP mRNA levels as determined by multiplexed quantitative PCR using B2M as housekeeping gene (ΔΔCt method). Cells were left untreated (NTC), treated with either 50 nmol/L PMA for 30 min followed by chase with EBM‐FBS with and without 10 μg/mL CHX (PMA and PMA+CHX) or treated with 4‐6 hours CM in presence or absence of CHX (CM and CM+CHX). (E‐G) Real‐time PCR quantitation of PLVAP relative mRNA levels 12 h post‐PMA (E) or 4‐6 hours CM (F, G) treatment in presence of inhibitors MAPK and PI3K/Akt pathways at indicated doses. Inhibitors were as follows: MAPKs:MEK1/2/ERK1/2 (U0126 and PD98059), p38 (SB203580 and SB202190), JNK (tanzisertib, SP600125); PI3K (GDC0941, idelalisib and wortmannin) and Akt (GDC0068). (SEM, n > 6, *P < 0.05 vs PMA or CM alone)

To determine which VEGFR is required for PMA/CM mediated PLVAP upregulation pharmacologic inhibitors with different selectivity for VEGFR1, 2 and 3 (Table 1) were used. For both PMA (Figure 6A) or 4‐6 hours CM (Figure 6B), both VEGFR2 inhibitors cabozantinib and vandetanib decreased PLVAP protein levels at 24 hours after treatment at both 1 and 10 μmol/L, whereas larger VEGFR spectrum Axitinib (Axi) and SAR131676 (SAR) had a detectable effect only at 10 μmol/L. Similar results were obtained for PLVAP mRNA measured at 8 hours post 4‐6 hours CM treatment (Figure 6C). Moreover, cabozantib, the VEGFR2 selective inhibitor, significantly reduced PLVAP mRNA (>50%) at doses as low as 0.01 μmol/L. These data strongly suggest a role for VEGFR2 but not VEGFR1 signalling in PLVAP upregulation by PMA or CM.

Taken together, the above data demonstrate that while VEGF‐A/VEGFR2 signalling is important for PLVAP upregulation by PMA, the latter induces the transcription and translation of additional EC factors that are required for efficient PLVAP induction.

### 3.5 PLVAP upregulation by conditioned medium or VEGF requires further protein synthesis

Both CM treated HDMVECn (Figure 6D) or 40 ng/mL VEGF‐A‐treated HUVEC (not shown) were unable to increase PLVAP mRNA in presence of CHX arguing that further EC protein synthesis is required for PLVAP upregulation.

To determine if the proteins elicited by CM were secreted, we carried out a CM/CM experiment. First, we established that a 30 minutes 4‐6 hours CM pulse up‐regulated PLVAP mRNA in time‐dependent manner with the increase starting at 4 and peaking at 8‐12 hours (data not shown) at ~75% of the levels obtained with a 24 hours incubation with same CM. None of the CM/CM peaks was able to significantly up‐regulate PLVAP mRNA in naive HDMVECn after 24 hours incubation (Figure S1B), demonstrating that while protein synthesis is required for PLVAP upregulation by the 4‐6 CM, it most likely involves the synthesis of cytoplasmic signalling molecules/transcription regulators rather than secreted/shed molecules.

### 3.6 PMA up‐regulates PLVAP in MEK1/2‐dependent and p38‐, JNK‐ and PI3K/Akt‐independent manner

We next explored the role of signalling pathways downstream of VEGFR2 using pharmacological inhibition (Table 1). The ability of both PMA (Figure 6E) and 4‐6 hours CM (Figure 6F,H) to up‐regulate PLVAP mRNA was inhibited by the MEK1/2 inhibitors U0126 and PD98059 in dose‐dependent manner. No changes in PLVAP upregulation were observed in the presence of p38 MAPK inhibitors such as SB203580 or SB202190, or JNK inhibitors SP600125 and tanzisertib (Figure 6E‐F,H).

Furthermore, PI3K signalling inhibition with both pictilisib and idelalisib had no impact on PLVAP upregulation by either PMA (Figure 6E) or 4‐6 hours CM (Figure 6F,G). However, wortmannin, another potent pan‐PI3K inhibitor reduces PLVAP mRNA and protein upregulation but only at the larger dose of 10 μmol/L, which may represent an off‐target inhibition of other kinases. Finally, consistent with lack of impact of PI3K inhibition, signalling downstream of PMA or CM does not require Akt, a main PI3K downstream effector, as shown by the lack of inhibition of PLVAP upregulation by Ipatasertib doses of 1‐10 μmol/L.

In each experiment, the effectiveness of each inhibitor was confirmed by Western blotting on their ability to block or activate specific phosphorylation events within known signalling pathways in response to PMA at 15, 30 and 60 minutes in HDMVECn: U0126—Erk1/2 phosphorylation; SB203580 and SB202190—p38 phosphorylation; pictilisib, idelalisib, wortmannin and GDC0068—Akt1 phosphorylation (data not shown). Thus, while the upregulation of PLVAP by PMA requires VEGF, there is a clear requirement for MEK1/ERK1/2 signalling whereas p38, JNK, PI3Kα/δ/γ and Akt1‐3 are not required in this system.

## 4 DISCUSSION

Research on endothelial diaphragms has been hampered by a lack of in vitro model systems that faithfully replicate the in situ biogenetic signalling. With this long‐term goal in mind, we began characterizing the signalling events during the DAG agonist (PMA) induction of PLVAP and diaphragms in primary EC. This system offers the advantage of allowing the study of PLVAP induction and formation of fenestrae.

Our data show that PLVAP mRNA upregulation by PMA is dependent on de novo protein synthesis. These newly synthesized proteins could be secreted proteins, cytoplasmic signalling molecules and/or regulators of transcription. By optimizing conditions for CM transfer experiments, we showed that neosynthesis of secreted molecules are required for PLVAP upregulation. A 30 minute pulse treatment with PMA up‐regulated PLVAP mRNA as early as 2 hours with a peak induction between 8 and 12 hours after treatment in dose‐dependent manner and was similarly effective in upregulating PLVAP protein as 24 hours at the same dose. CM experiments showed the presence within 4‐8 hours after PMA treatment of newly synthesized, heat labile proteins that are able to induce PLVAP mRNA and protein in PMA naive EC. The CHX experiments and the relative lack of PLVAP‐inducing “activity” in the CM collected 1 hour after PMA treatment strongly argue that transcription and protein synthesis is required to generate the factor(s) responsible for PLVAP upregulation. It also ruled out the secretagogue effect of PMA^50^ or leakage of PMA from donor cells into the CM.

Conditioned medium fractionation by ultracentrifugation showed that most of the PLVAP‐inducing factors are soluble proteins. We have found, however, that the particulate fraction of the CM (expected to contain exosomes) is also able to induce PLVAP upregulation albeit at lower levels. Further proteomic experiments should elucidate whether the PLVAP‐inducing proteins in the soluble and particulate fractions of the CM are the same or not.

Heparin depletion experiments demonstrated that multiple factors in the CM may be involved in PLVAP upregulation by PMA with most but not all of these factors bind heparin.

We were able to rule out a role for chemokines, known to bind heparin, as PT was unable to inhibit PLVAP upregulation by the 4‐6 and 6‐8 hours CM at a dose widely accepted in the literature as effective.

Among the PMA‐induced growth factors known to bind heparin, a survey of the literature yielded VEGF‐A as a candidate. VEGF‐A signalling via VEGFR2 up‐regulates PLVAP in single donor HUVEC.^30^ While we confirmed the rapid upregulation by PMA of VEGF‐A mRNA peaking at 60‐90 minutes after treatment, no significant VEGFR2 mRNA increase was observed in HDMVECn up to 24 hours after treatment. However, HDMVECn already express substantial levels of VEGFR2. Additionally, PMA up‐regulated VEGFR1 (Flt 1) as early as 2 hours after treatment with a peak at 4‐8 hours, although the significance of this increase to the overall economy of VEGF‐A signalling in this system is unclear.

While VEGF‐A165 elicited low levels of PLVAP mRNA and protein in HDMVECn, PMA or post‐PMA CM has a >10‐fold greater ability to up‐regulate PLVAP. Anti‐VEGF‐A antibody blockade and VEGF‐A mRNA knockdown with siRNA partially inhibits the ability of PMA or post‐PMA CM to up‐regulate PLVAP, arguing that VEGF plays a role in PLVAP upregulation. Addition of exogenous 40 ng/ml VEGF (a dose widely accepted in the literature as activating) to PMA or CM did not increase PLVAP transcription at different time points and protein levels at 24 hours, suggesting that: (a) VEGF‐A signalling is an early step within a sequential signalling pathway leading from PMA to PLVAP upregulation; or, (b) VEGF‐A signalling synergizes with/is additive to other signalling factors induced by PMA and by the time these factors are up‐regulated, VEGF‐A levels are already saturating, hence the lack of synergistic or additive effect between VEGF‐A and PMA or CM. While not particularly enhancing either scenario, translation arrest experiments demonstrate that both VEGF‐A and CM require downstream de novo protein synthesis to be able to up‐regulate PLVAP. Finally, VEGFR2 is thought to transduce all known effects of VEGF‐A^51^ and was implicated in VEGF upregulation of PLVAP.^30^ The pharmacological inhibition results strongly support a role for VEGFR2 in PLVAP upregulation by PMA and CM. The rather sizeable effects of VEGF‐A knockdown and VEGFR2 inhibition on PLVAP transcription lend support to a model in which VEGF/VEGFR2 signalling is an early event in a series of signalling events leading to PLVAP upregulation or a necessary synergistic partner to other factors. Additionally, our results may also implicate VEGFR3 (Flt 4) in PLVAP upregulation by PMA and 4‐6 hours CM and this relationship will be further explored in future work.

Given the role of VEGF/VEGFR2 signalling in PLVAP upregulation by PMA, there is apparent conflict between earlier data showing that PMA upregulation of PLVAP was MEK1‐dependent^16^ whereas PD98059 (a MEK1 inhibitor) had little effect on PLVAP mRNA and protein upregulation at 48 hours post VEGF‐A treatment.^30^ Using the same two inhibitors (U0126 and PD98059) we confirmed our earlier findings that PMA up‐regulates PLVAP in MEK1/ERK1/2—dependent manner and we further show the same to be true for PLVAP upregulation by the post‐PMA CM. A simple explanation of this discrepancy would be that the other secreted factors required for PLVAP upregulation by PMA signal through MEK1/ERK1/2 pathway. Notwithstanding the cell culture differences between our work and earlier reports regarding cell starvation medium and length, cell type, composition of the stimulation medium and especially length of stimulation, these differences could be technical (ie PD98059 requires higher doses for MEK 1 inhibition and is rather unstable) and the case is that VEGF‐A does induce PLVAP upregulation via ERK1/2. VEGF‐A is known to activate the Raf/MEK/ERK pathway in human ECs (reviewed in^51^). Unfortunately, due to the rather inefficient upregulation of PLVAP by VEGF‐A in our cells we were unable to reliably investigate the effect of these inhibitors on PLVAP upregulation by VEGF‐A. In any case, the Erk1/2 downstream effectors involved in PLVAP upregulation would be interesting to elucidate in the future.

Plasmalemma vesicle associated protein has also been shown to be regulated by VEGF‐A/VEGFR2 signalling in p38 MAPK‐dependent manner although no known downstream targets of p38 were found to be involved.^30^ Quantitative mRNA analysis (data not shown) demonstrated that HDMVECn express all the p38 isoforms (p38α/MAPK14, p38β/MAPK11, p38γ/MAPK12, p38δ/MAPK13), with p38γ/MAPK12 being the least abundant. Nevertheless, our results show no inhibition of PLVAP mRNA upregulation at 8 and 24 hours post either PMA or CM treatment by two widely used pan p38 inhibitors, despite our demonstration that the same doses of these inhibitors block p38 MAPK phosphorylation in response to PMA. Both inhibitors are active against all p38 isoforms with IC50 in the nanomolar range and the doses used were 2‐3 orders of magnitude larger. However, while our data do not support a role for p38 signalling in PMA or CM induced PLVAP upregulation, these results are puzzling given the role of VEGF/VEGFR2 signalling in this process.

Others have suggested that VEGF‐A regulates PLVAP expression in PI3 kinase‐dependent manner.^30^ HDMVEC express all four isoforms of p110 (α/PIK3CA, β/PIK3CB, γ/PIK3CG and δ/PIK3CD) and the respective p85/p55/p150 regulatory subunits (PIK3R1‐4). Using novel and more selective PI3K pharmacological inhibitors that are in clinical trials such as pictisilib (highly selective for PI3Kα/δ and 11‐25 fold lower selectivity on PI3Kβ/γ) and idelalisib (selective for PI3Kγ>δ), we show that PI3K inhibition does not inhibit PLVAP upregulation by PMA or CM. Accordingly, the inhibition of Akt1‐3, a major downstream target of PI3K, does not impact PLVAP upregulation. To note, wortmannin, a pan PI3K inhibitor, is partially effective at the larger concentration of 10 μmol/L either suggesting off‐target effects or a role for p110β, which is not covered as well by the other inhibitors used. However, the latter is less likely as the dose of pictisilib we used is several orders of magnitude larger than the IC50 for p110β.

In summary, we find that PLVAP upregulation by PMA requires de novo synthesis of multiple secreted proteins that act in an autocrine manner. One of the soluble factors involved is VEGF‐A acting through VEGFR2. The signalling is dependent on MEK1/ERK1/2 and independent of p38, JNK, PI3K and Akt1‐3. Further transcriptomic and proteomic studies should identify the factors (or combinations thereof) contributing to PLVAP regulation.

## ACKNOWLEDGEMENTS

This work was supported from National Institutes of Health grant 1R01GM120592 and American Heart Association grant 16GRNT27260362 to RVS. NCCC DartLab and Microscopy Shared Resources are supported by NCI Cancer Center Support Grant 5P30CA023108‐37. We would like to thank Yan Xu for technical assistance in early stages of this project, N. Shworak for advice and C. Carriere for critically reading the manuscript.

## CONFLICT OF INTEREST

The authors confirm that there are no conflicts of interest.

## AUTHOR CONTRIBUTIONS

BJH, DT and RVS performed and designed experiments. BJH and RVS wrote the paper.
